# Prevalence and Characteristics of Hepatic Hemangioma Associated with Coagulopathy and Its Predictive Risk Factors

**DOI:** 10.3390/jcm11154347

**Published:** 2022-07-26

**Authors:** Shigeo Maruyama, Tomomitsu Matono, Masahiko Koda

**Affiliations:** 1 Maruyama Medical Clinic, Aioimacho 3921, Hamada 697-0034, Shimane, Japan; shigeo.maruyama0304@gmail.com; 2 St. Mary’s Hospital, Nibuno 650, Himeji 670-0801, Hyogo, Japan; matomato@himemaria.or.jp; 3 Hino Hospital, Nota 332, Hino 689-4504, Tottori, Japan

**Keywords:** hepatic hemangioma, coagulopathy, coagulation factor, hemangioma-related complication

## Abstract

Background: Knowledge of the relationships between hepatic hemangiomas and coagulopathy and the risk factors for hemangiomas is lacking. The aim of this study was to investigate the prevalence and characteristics of hepatic hemangiomas associated with coagulopathy, elucidate the causes of coagulopathy, and identify the predictive factors for hemangioma-related complications. Methods: In 281 consecutive patients with hepatic hemangiomas, we performed ultrasonography and conducted serum laboratory tests for liver function and six coagulation factors, i.e., platelets, as well as five coagulation fibrinolytic markers (prothrombin time (PT), fibrinogen, thrombin-antithrombin III complex (TAT), d-dimer, and fibrin and fibrinogen degradation products (FDP)) as indicators of coagulation disorder. Results: Among 281 patients, 56 (19.9%) had abnormal coagulation factors. Abnormal values of d-dimer were most frequently found among the six coagulation factors. The number of abnormal coagulation factors was significantly correlated with tumor size, M2BPGi, and HDL cholesterol, among which tumor size was the most significant independent predictor of the number of abnormal coagulation factors. Conclusions: The prevalence of hepatic hemangiomas associated with coagulopathy was relatively high and became more frequent with increases in tumor size. Predictive factors of hemangioma-related complications were found to be a tumor size of >5 cm in diameter and coagulopathy, especially the elevation of d-dimer.

## 1. Introduction

Hepatic hemangiomas are well known as the most common benign tumors of the liver and are rarely complicated by blood coagulopathy, although coagulation disorders, including thrombocytopenia and hypofibrinogenemia, are sometimes associated with hemangiomas [1,2,3,4,5]. Kasabach–Merritt syndrome (KMS) is a rare complication of giant hepatic hemangiomas that is characterized by thrombocytopenia and consumption coagulopathy and is considered an absolute indication for surgical intervention [6,7,8]. However, knowledge of the prevalence and characteristics of hepatic hemangiomas associated with coagulopathy and the risk factors for hemangioma is limited. We previously investigated the correlation between tumor size or the internal echo pattern of hepatic hemangiomas and coagulation factors, and concluded that differences in the tumor size and echogenicity were caused by intra-tumoral thrombosis and subsequent hemorrhage [9]. The aim of this study was to investigate the prevalence and characteristics of hepatic hemangiomas associated with coagulopathy, to elucidate the causes of coagulopathy, and to identify the predictive factors of hemangioma-related complications.

## 2. Materials and Methods

### 2.1. Patients

This study was approved by the ethics review board of Tottori University Hospital (approval number: 18A023) and the ethics committee of Hino Hospital (approval number: 2018-4). Among the abdominal ultrasonography (US) examinations performed at our hospitals, Hino Hospital, and Tottori University Hospital, Japan, between January 2016 and September 2020, a total of 281 patients were diagnosed with hepatic hemangiomas and were consecutively enrolled in this study after providing informed consent. Patients with infectious diseases, liver cirrhosis, or malignant tumors were excluded from the study. Patients with abnormal values of thrombin–antithrombin III complex (TAT) (>3.0 ng/mL), d-dimer (>1.0 µg/mL), and fibrin and fibrinogen degradation products (FDP) (>5.0 µg/mL) were referred for cardiovascular medicine consultation to prevent complications of thrombosis, such as deep vein thrombosis (DVT) or lung thrombosis. No patients were treated with anti-coagulation therapy.

### 2.2. Methods

Hepatic hemangiomas were diagnosed by US and multiphase contrast-enhanced helical computed tomography (CT). In most hemangiomas (diameter ≤ 20 mm), the final diagnosis was based on US features, clinical observation, and negative biochemical results. Relatively large lesions (diameter > 20 mm) were diagnosed using both US and CT. Abdominal US was performed in all patients, and CT was performed in 98 patients. The US findings were reviewed and the following were recorded: total number, size, echogenicity, as well as the location of hepatic hemangiomas, diameter of the portal vein, and the spleen index. In the case of multiple hemangiomas, the tumor with the largest diameter was selected for analysis. All patients were examined by US examiners who had 30 years of experience. Using the category described in a previous study of giant hemangiomas [1], we divided the hepatic hemangiomas into three groups according to the maximum diameter: small group (<20 mm), medium group (20–40 mm), and large group (>40 mm). The internal echo pattern was classified into two groups: homogeneous group (homogeneous hyperechoic, homogeneous hypoechoic, and isoechoic), and mixed group (mixed hyperechoic and mixed hypoechoic) [10].

Routine laboratory tests were performed using automated methods. Blood platelet counts in the range of 14–38 × 10^4^/μL were considered normal. Prothrombin activity was determined by the coagulation method using the one-stage prothrombin-time assay (Japan Clinical Laboratory, Kyoto, Japan), with values in the range of 70–130% considered normal. Fibrinogen was determined by a rapid physiological coagulation technique using the clotting method (Japan Clinical Laboratory), with values in the range of 180–400 mg/dL considered normal. Plasma TAT concentration was determined by a chemiluminescent enzyme immunoassay (Special Reference Laboratory, Tokyo, Japan), with a TAT level of <3.0 ng/mL considered normal. The concentrations of d-dimer and FDP were determined by a latex immunoturbidimetric assay (Japan Clinical Laboratory). Normal d-dimer and FDP levels are <1.0 µg/mL and <5.0 µg/mL, respectively.

On the basis of 6 coagulation factors, i.e., platelets and 5 coagulation fibrinolytic markers (PT, fibrinogen, TAT, d-dimer, and FDP), we divided the patients into three groups according to the number of abnormal factors related to platelets and coagulation fibrinolytic markers. The A group consists of patients with normal values for platelets, PT, fibrinogen, TAT, d-dimer, and FDP. The B group consists of patients with abnormal values for only 1 of the above 6 factors. The C group consists of patients with abnormal values for two or more factors.

### 2.3. Statistical Analysis

All measurements are expressed as the mean ± standard deviation (SD). Statistically significant differences among the three size groups and the two echo pattern groups were determined using Student’s t-test or one-way analysis of variance with Kruskal–Wallis testing and Dunn’s test as a post hoc test. Categorical variables were analyzed using the chi-square test. Correlation analysis was carried out by univariate linear regression analysis. The multivariate forward stepwise regression analysis was used to identify independent factors related to the tumor size of hepatic hemangiomas. The data were analyzed using Stat Flex version 6.0 (Artech Co., Ltd., Osaka, Japan). A *p*-value of < 0.05 was considered statistically significant.

## 3. Results

### 3.1. Patients Characteristics

**Figure 1:**
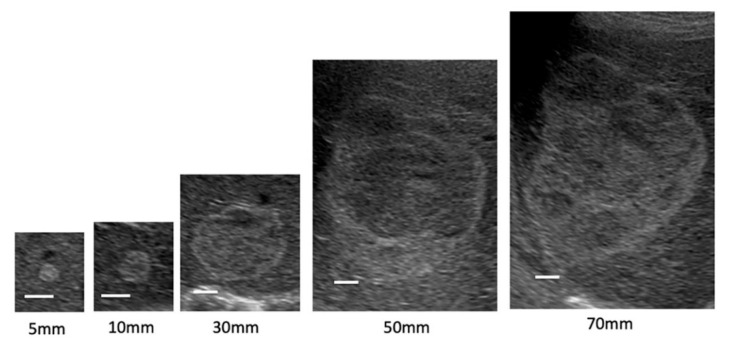
Internal echo pattern according to size of hemangiomas. White bars indicate a length of 10 mm.

Figure 1 shows the internal echo patterns of hemangiomas according to the tumor diameter. Most hemangiomas with a diameter of ≤20 mm were homogeneous. As the size increased, the internal echo pattern became the mixed echo type.

Among the 283 patients with hepatic hemangiomas, 2 patients who had high values of protein induced by vitamin K absence-2 (PIVKA-II) were excluded because differentiation from malignant tumors was required. A total of 281 patients were enrolled in the present study. The laboratory findings of the enrolled patients are shown in Table 1. There were 98 men and 183 women (male:female ratio, 1:1.9), with a median age of 54 years (range, 23–89 years). All patients, except one with abdominal distension, were asymptomatic and had their hemangiomas discovered by health examination or incidental radiological studies. At the time of diagnosis, serum laboratory tests were normal in 90.7% (255/281) of patients. Twenty-six patients had a slight elevation in alanine aminotransaminase (ALT), γ-glutamyl transpeptidase (GGT), or alkaline phosphatase (ALP). No patients received surgical treatment.

The US findings are shown in Table 2. The sonographic appearance of hemangiomas was hyperechoic in 209 (74.4%), hypoechoic in 2 (0.7%), and isoechoic in 1 patient (0.4%). The lesions presented a mixed echo pattern in 69 patients (24.5%). Among the patients, 190 (67.6%) were in the small group, 67 (23.9%) in the medium group, and 24 (8.5%) in the large group. The lesions were located in the right liver lobe in 247 (87.9%), in the left liver lobe in 32 (11.4%), and bilaterally in 2 (0.7%) patients. Single hemangiomas were detected in 240 patients (85.4%), while the remaining 41 patients (14.6%) had multiple tumors. The median portal diameter and spleen index were 10.6 mm and 1293 mm^2^, respectively.

### 3.2. Association among Hemagioma Size, Clinical Parameters, and Coagulation Factors

Table 3a shows the relationship between tumor size and clinical parameters in patients with hepatic hemangiomas. There was no significant difference in terms of mean age or sex ratio among the three groups. Albumin concentration was significantly lower in the large group than in the other groups (*p* < 0.01), but other biochemical values except albumin, blood urea nitrogen (BUN), high-density lipoprotein (HDL) cholesterol, and hemoglobinA1c (HbA1c) were not significantly different among the three groups. Hemoglobin concentration was significantly lower in the large group than in the other groups (*p* < 0.05). Mac-2 binding protein glycosylation isomer (M2BPGi) and PIVKA-II levels were significantly elevated in the large group (*p* < 0.0001 and *p* < 0.001, respectively). Almost all tumors (99.5%) in the small group were homogenous. In the medium group, a homogenous pattern was seen in 34.3% (23/67) of hemangiomas and a mixed echo pattern in 65.7% (44/67). All hemangiomas in the large group had a mixed echo pattern. Portal vein diameter and spleen index were significantly elevated in the large group (*p* < 0.0001 and *p* < 0.001, respectively).

Table 3b shows the relationship between the tumor size and coagulation markers and abnormal number of coagulation factors in patients with hepatic hemangiomas. Platelet counts (*p* < 0.0001) and fibrinogen levels (*p* < 0.01) were significantly lower in the large group than in the small and medium groups. PT was similar among the three groups, but TAT, d-dimer, and FDP values were significantly elevated in the large group (all *p* < 0.0001). An abnormal number of coagulation factors was significantly higher in the large group than in the small and medium groups (both *p* < 0.0001). Of the 24 patients in the large group, all 16 patients with tumors larger than 50 mm in diameter had two or more abnormal coagulation factors and the average number of abnormalities was 2.8 factors (range, 2–5). Meanwhile, eight patients with tumors 40 mm or more and less than 50 mm in size had 1.9 abnormal coagulation factors (range, 1–3), and there was a significant difference between the above two tumor size groups (*p* < 0.05) (data not shown).

Table 4 shows the clinical characteristics of 281 patients categorized according to number of abnormal coagulation factors (A, B, and C groups). There was no significant difference in mean age or sex ratio among the three groups. Albumin and HDL cholesterol concentrations were significantly lower in the C group than in the other groups (both *p* < 0.01), but all biochemical values except albumin and HDL cholesterol were not significantly different among the three groups. Hemoglobin concentration was similar in the three groups. M2BPGi and PIVKA-II levels were significantly elevated in patients in the C group (*p* < 0.0001 and *p* < 0.001, respectively). The tumor size in the C group was significantly larger than in the A and B groups (both *p* < 0.0001). In the A and B groups, a homogenous pattern was seen in 88.0% and 43.8% of hemangiomas, and a mixed echo pattern in 12.0% and 56.2%, respectively. All hemangiomas in the C group had a mixed echo pattern. These results show that the internal echo pattern became mixed with an increase in the number of abnormal coagulation factors. The portal vein diameter and spleen index were significantly higher in the C group than in the other groups (*p* < 0.0001 and *p* < 0.001, respectively).

Table 5 shows the occurrence rate of abnormal coagulation factors (platelet, PT, fibrinogen, TAT, d-dimer, and FDP). The occurrence rate of abnormal coagulation factors was 1.8% (5/281) in platelets, 0.7% (2/281) in PT, 2.5% (7/281) in fibrinogen, 8.2% (23/281) in TAT, 16.0% (45/281) in d-dimer, and 4.6% (13/281) in FDP, i.e., abnormal d-dimer values were most frequently found. Among the 281 patients, 56 (11.9%) had abnormal coagulation factors, one of which was found in 32 patients (11.4%), whilst two factors were found in 13 (4.6%), three factors were found in 8 (2.8%), four factors were found in 2 (0.7%), and five were found factors in 1 (0.4%).

Table 6 shows the multiple regression analysis of variables associated with the number of abnormal coagulation factors in patients with hepatic hemangiomas. The number of abnormal coagulation factors was significantly correlated with the tumor size, M2BPGi, and HDL cholesterol. Tumor size was the most significant independent predictor of the number of abnormal coagulation factors.

Table 7 shows a comparison of clinical parameters in patients with and without chronic liver disease as an underlying disease. The objective of this table is to investigate the differences of various parameters by the presence or absence of liver disease and to elucidate the involvement of liver disease for hepatic hemangiomas. Forty-one patients had chronic liver disease as an underlying disease (details are described in Table 1). The mean age of patients with chronic liver disease was significantly higher than in those without (*p* < 0.001). The sex distribution was similar in both groups. Values of ALT (*p* < 0.01) and GGT (*p* < 0.05) were significantly higher, and platelet counts (*p* < 0.01) and fibrinogen levels (*p* < 0.05) were significantly lower in patients with chronic liver disease than in those without. Values of TAT, d-dimer, and FDP were similar in both groups. The M2BPGi level and portal vein diameter were significantly higher in patients with chronic liver disease than in those without (*p* < 0.001 and *p* < 0.05, respectively). The tumor size and number of abnormal coagulation factors were slightly but not significantly increased in patients with chronic liver disease.

Table 8 shows a comparison of clinical parameters in patients with and without atherosclerotic disease. The objective of this table is to investigate the differences of various parameters by the presence or absence of atherosclerotic disease and to elucidate the involvement of atherosclerosis for hepatic hemangiomas. Hypertension, dyslipidemia, and diabetes mellitus were selected as representative atherosclerotic diseases. Sixty-nine patients had atherosclerotic disease, and some had two or more diseases (details are given in Table 1). The sex distribution was similar in both groups, but the mean age in patients with atherosclerotic disease was significantly higher than in those without (*p* < 0.0001). Values of BUN, creatinine, triglyceride, glucose, and HbA1c were significantly higher, while HDL levels were significantly lower in patients with atherosclerotic disease than in those without. Platelet count, fibrinogen level, and values of TAT and FDP were similar in both groups, while the d-dimer value was significantly higher in patients with atherosclerotic disease than in those without (*p* < 0.05). Values of M2BPGi and PIVKA-II were significantly higher in patients with atherosclerotic disease than in those without (*p* < 0.05 and *p* < 0.01, respectively). Tumor size and abnormal number of coagulation factors were similar in both groups.

## 4. Discussions

Hepatic hemangiomas are rarely complicated by blood coagulopathy, although coagulation disorders, including thrombocytopenia and hypofibrinogenemia, are sometimes associated with hemangiomas [1,2]. The combination of thrombocytopenia, hypofibrinogenemia, and the activation of the fibrinolytic system implicates a situation of continuous coagulation followed by fibrinolysis within the vessels of hemangiomas. The bleeding diathesis associated with giant hemangiomas is best interpreted as intravascular coagulation and subsequent fibrinolysis [11]. Platelets and fibrinogen implicate coagulation, while TAT, d-dimer, and FDP reflect coagulation and fibrinolysis. Platelet counts and fibrinogen levels decrease with increasing TAT, d-dimer, and FDP levels in patients with hemangiomas [9,12]. In the present study, patients with larger hemangiomas had significantly lower platelet counts and fibrinogen levels, and the prominent elevation of d-dimer and FDP coexisted with increasing levels of TAT (data not shown), suggesting that the formation of thromboembolism and fibrinolysis occur repeatedly within the vessels of hemangiomas. The clotting–fibrinolysis theory within hemangiomas [11] was apparently proven by our study.

PT assesses plasma coagulation function and screens the extrinsic or tissue factor-dependent pathway. PT also evaluates the common coagulation pathway involving all the reactions that occur after the activation of factor X. Although the reduction in PT suggests the possibility of coagulation accelerators such as thrombosis, it has little clinical significance [13]. Actually, our results demonstrated that PT did not significantly differ among patients with various types of tumors and abnormal PT values were found in only 2 (0.7%) of 281 patients; therefore, we speculated that PT was not one of the important evaluations of coagulation disorders complicated by hemangiomas.

Fibrinogen is converted into fibrin by activated thrombin and is a key protein in the coagulation pathway and clot formation, and supports platelet aggregation, which represents the final step of the coagulation cascade. Platelets are another crucial player in the coagulation system [14]. A previous study reported that plasma fibrinogen levels were correlated with platelet counts [15]. Our previous and present studies demonstrated that patients with larger lesions or mixed echo pattern had significantly lower platelet counts and fibrinogen levels [9]. We speculated that two possible causes in the decrease in fibrinogen levels and platelet counts complicated with hemangiomas were as follows, respectively: (1) enhanced thrombotic formation through platelet aggregation, (2) increase in consumption of fibrinogen caused by increased activation of fibrinolysis and; (1) entrapment of platelets in vascular spaces of hemangioma and activation of coagulation, (2) consumption of platelet in addition to ongoing fibrinolysis.

Previous research using 51Cr-platelets or 125I-fibrinogen [11,16,17,18] demonstrated the rapid disappearance of radioactive platelets and fibrinogen from circulation in hemangioma patients. Low platelet counts and fibrinogen levels are considered to be the result of intravascular coagulation in the hemangioma, and it is concluded that platelets and fibrinogen were consumed or degraded in the hemangioma [18]. The consumption of platelets and fibrinogen, in addition to ongoing fibrinolysis, results in intra-tumoral thrombosis or hemorrhage and the subsequent enlargement of the hemangioma [9,12].

TAT is generated in the process of thrombin formation and is direct evidence of the increased activation of coagulation [19]. Therefore, the TAT level reflects the early phase of thrombosis and is useful as a predictor of the prethrombotic state. Elevated TAT reflects the activation of the coagulation system and indicates a hypercoagulable state, and thrombosis can be easily formed in such a situation together with the stasis of blood flow occurring within the vessels of hemangiomas [19]. d-dimer, a product of fibrin degradation, reflects both the activation of fibrinolysis and the severity of the hypercoagulable state. The elevation of d-dimer can result from the marked activation of blood coagulation, leading to the massive formation of intravascular thrombosis. Accordingly, the increased plasma levels of d-dimer are considered to be the result of thrombus formation and reflect fibrinolysis [20]. FDP consists of both fibrin and fibrinogen-degradation products and is an important marker of coagulopathy such as disseminated intravascular coagulation (DIC) and DVT [21], and FDP levels are often closely correlated with d-dimer [22]. Thus, elevated FDP, including d-dimer, reflects a thrombotic state and suggests the presence of venous thromboembolism [2]. TAT, d-dimer, and FDP are all increased in the acute phase of thrombosis, while in the chronic phase of thrombosis TAT is slightly increased, whereas d-dimer and FDP are markedly increased [23,24]. Since d-dimer and FDP reflect secondary fibrinolysis after clot formation, they may not be useful as early predictive markers for thromboembolism. In contrast, the TAT level is direct evidence of the increased activation of coagulation and reflects the early phase of thrombosis. The half-life of TAT is short, whereas that of d-dimer is long. Thus, TAT levels increase rapidly and diminish relatively soon after the onset of thromboembolism [19,23,24], while the plasma levels of d-dimer are further increased after the onset of thromboembolism [24]. Therefore, measurements of both d-dimer and TAT may be recommended. Although FDP levels can be closely correlated with d-dimer, plasma concentrations of FDP diminish relatively faster than those of d-dimer [24]. In the present study, almost all patients with a prominent elevation of d-dimer and FDP coexist with increased levels of TAT, suggesting that the hypercoagulable state is present, and subsequently, the formation of thromboembolism and fibrinolysis occurs repeatedly within the vessels of hemangiomas.

KMS is well known as a giant hepatic hemangioma characterized by consumption coagulopathy. The pathophysiology of KMS developing secondarily to hepatic hemangioma is considered as the entrapment of platelets in the vascular spaces of the hemangioma, and activation of coagulation and fibrinolysis mechanisms [6]. The consumption of platelets and coagulation factors, as well as ongoing fibrinolysis, results in the intra-tumoral bleeding and enlargement of the hemangioma [7]; consequently, hemangiomas may lead to KMS during a certain period of life. PT is typically prolonged, fibrinogen is reduced, and FDP and d-dimer can be elevated [8]. We previously investigated the correlation between tumor size and internal echo pattern of hepatic hemangiomas and coagulation factors and concluded that differences in the tumor size and echogenicity were caused by intra-tumoral thrombosis and subsequent hemorrhage [9] and were associated with coagulation factors, especially TAT, d-dimer, and FDP. From the above, we can speculate that coagulation factors may play a part in molecular mechanisms that contribute to hepatic hemangiomas and may be related to its pathogenesis in the process of the coagulation pathway.

Our results show that abnormal d-dimer values were most frequently found among the six coagulation factors. d-dimer has been reported to be the most representative predictor for venous thromboembolism and is elevated in patients with DIC, DVT, pulmonary embolism (PE), acute myocardial infarction, and thrombotic thrombocytopenic purpura [24,25,26,27]. Plasma concentrations of d-dimer were significantly high in patients with all types of thrombosis [26]. The elevated d-dimer level indicates a high risk of thrombosis and is thus considered to be useful as a negative predictor for thrombosis [28]. In Europe and North America, d-dimer concentrations of less than 0.5 μg/mL are considered to exclude DVT/PE [25,26,27,28], although some d-dimer kits that are frequently used in Japan have a different cut-off value (1.2 μg/mL) for the exclusion of DVT/PE [27]. This divergence in d-dimer values is considered to be a result of the standardization of the d-dimer assay used in Japan. Furthermore, the d-dimer assay results in Japan might be 2-fold higher than the results of assays commonly used in Europe and North America [25,26]. Some previous reports indicated that more than 50% of patients with d-dimer values >3.0 μg/mL had some thrombosis [24,26], and a d-dimer concentration of 4.8 μg/mL was considered to be an appropriate cut-off value for the diagnosis of venous thromboembolism with different assays [27]. Our present study demonstrated that abnormal d-dimer values were most frequently found among the 6 coagulation factors and all 16 patients with tumors larger than 50 mm in diameter had abnormal d-dimer values; the mean d-dimer level was 2.6 μg/mL, and ranged from 1.2 to 4.9 μg/mL. d-dimer levels greater than 5.0 μg/mL were not found in any of these patients. From our results and previous reports, we speculated that d-dimer was the most representative predictor for venous thrombosis, and a d-dimer concentration of more than 5.0 μg/mL is estimated as the value leading to the massive formation of thrombosis within the vessels of hemangiomas and is considered to be one of the predictive factors for hemangioma-related complications.

PIVKA-II is an abnormal form of prothrombin in which some or all ten γ-carboxyglutamic acid (Gla) residues remain as glutamic acid (Glu) residues and has been largely used as an indicator of blood coagulation abnormality or vitamin K deficiency [29]. The appearance of PIVKA-II correlates with a reduction in the products of prothrombin resulting from vitamin K deficiency [29] and therefore the elevation of PIVKA-II is not directly related to the coagulation disorder caused by the abnormality of PT itself. Actually, the elevation of PIVKA-II levels seen in our study might not have important significance as an indicator for coagulopathy complicated with hemangiomas; furthermore, our results demonstrated that PT values were not significantly different among patients with various types of hemangiomas and were not significantly correlated with PIVKA-II values (data not shown). These findings indicate that abnormal values of PIVKA-II are not related to coagulopathy complicated with hemangiomas.

Our results show that M2BPGi [30,31], one of the biochemical markers of liver fibrosis, was significantly elevated along with an increasing number of abnormal coagulation factors, and was one of the independent factors of coagulopathy associated with hemangiomas. Hemangiomas can undergo degeneration and fibrous replacement within tumors [32] involuting with regressive changes such as thrombosis, necrosis, calcification, and/or fibrosis [5]. Fibrosis noted around the hemangiomas might be caused by regional circulatory disorders in the peripheral liver parenchyma triggered by thrombus formation within tumors [33] and blood flow disturbances caused by tumor compression effects with increasing tumor size [34]. Fibrous degeneration and replacement within hemangiomas and progressive and extensive fibrosis surrounding tumors resulted in the focal fibrosis of the liver. Our results demonstrate that M2BPGi levels are correlated with platelets (*p* < 0.0001), albumin (*p* < 0.0001), PT (*p* < 0.05), ALT (*p* < 0.0001), and age (*p* < 0.0001) (data not shown), as shown in previous studies [31,35], suggesting that the elevation of M2BPGi might be caused by the development of focal liver fibrosis within and surrounding the hemangiomas. M2BPGi levels were closely related with the fibrosis stage of chronic liver disease [36]. Actually, in the present study, values of M2BPGi in 41 patients with chronic liver disease as an underlying disease were significantly higher than those in 240 patients without chronic liver disease. In addition, chronic liver disease as an underlying disease was seen in 27 of 225 patients (12.0%) in the A group, 8 of 33 (24.2%) in the B group and 6 of 23 (26.1%) in the C group, and there was a significant difference among the three groups (*p* < 0.05). These findings suggest that the M2BPGi values in each group might be influenced by the presence of chronic liver disease as an underlying disease. However, mean M2BPGi values were 0.95 COI in the large group or 0.96 COI in the C group, which were within normal values, and were lower than 1.00 COI, which was the optimal cutoff value for patients with fibrosis grade F1 [30,31], suggesting that the elevation of M2BPGi seen in our study might not be pathologically significant compared to the degree of fibrosis in patients with chronic liver disease. Therefore, M2BPGi in itself might be not directly involved with coagulopathy associated with hemangiomas. However, the hemangiomas in our study were not surgically resected and a pathologic correlation was not available; therefore, further studies are required to evaluate the exact relationship between focal liver fibrosis and coagulation disorders associated with hemangiomas.

The present study showed that HDL cholesterol was significantly decreased coincident with an increased number of abnormal coagulation factors, and was one of the independent factors of coagulopathy associated with hemangiomas. HDL cholesterol is one of the parameters in dyslipidemia and is associated with atherosclerosis. Sugiura et al. investigated the hypothesis that serum M2BP concentrations may change during the atherosclerotic process, demonstrating that M2BPGi levels were associated with increased arterial stiffness and subclinical atherosclerosis. Moreover, their study showed that a change in serum M2BP concentrations was significantly correlated with changes in HDL and LDL cholesterol [36,37,38]. Our results also demonstrate that M2BPGi levels were significantly correlated with total cholesterol (*p* < 0.01) and HDL cholesterol (*p* < 0.001) values (data not shown). Moreover, our study compared patients with and without atherosclerotic disease, and HDL cholesterol levels were significantly lower and M2BPGi concentrations were significantly higher in patients with atherosclerotic disease than in those without. These findings indicate that serum HDL cholesterol levels were associated, at least in part, with atherosclerotic risk factors complicated by hemangiomas. D-dimer was significantly higher in patients with atherosclerotic disease than in those without, although the hemangioma size was similar in both groups, suggesting that atherosclerosis might be in some way related to the elevation of D-dimer; however, it might not be involved in the change in size of hepatic hemangiomas. There have been no previous reports regarding the causal association between hemangiomas and atherosclerosis. Based on our results and the available literature, we speculated that atherosclerosis might be involved in some way with coagulopathy associated with hemangiomas and has the potential to cause the occurrence of blood coagulation disorder; however, further studies are required to evaluate the possible association between atherosclerosis and coagulopathy related to hemangiomas.

We examined the independent factors among the clinical parameters for the number of abnormal coagulation factors and found that tumor size was the most important independent factor of coagulopathy associated with hemangiomas. We previously demonstrated that the size of hemangiomas was closely associated with coagulation factors and the increase in tumor size was caused by intra-tumoral thrombosis and subsequent hemorrhage [9]. Our present study showed that platelet counts and fibrinogen levels were significantly reduced and values of TAT, D-dimer, and FDP were significantly increased with significant correlations with tumor size, and the number of abnormal coagulation factors was significantly elevated with the increase in tumor size. As hemangiomas increased in tumor size, the coagulation disorder was further aggravated. Hemangiomas are conventionally categorized as giant at a diameter of greater than 4 cm [1,2]. However, other sizes, such as >5 cm, >8 cm, or >10 cm, are subsequently specified to categorize a hepatic hemangioma as giant [1]. Some authors have suggested that hepatic hemangiomas with a diameter of >5 cm can be referred to as giant hemangiomas and that tumor size could be a decision factor for medical intervention modalities [1,2,3,4,39]. Others have also suggested that giant hemangiomas, defined by a diameter larger than 5 cm, might give rise to mechanical complaints requiring intervention [40,41]. Our present study demonstrated that the mean tumor size in patients with two or more abnormal coagulation factors was 59.5 mm, which was significantly larger than in patients with one factor or less. In the large group with a tumor size of ≥40 mm, all patients with tumors ≥50 mm in diameter had two or more abnormal coagulation factors and the average number of abnormal factors was 2.8 (range, 2–5), while patients with tumors of <50 mm had 1.9 factors (range, 1–3), showing a significant difference between them, suggesting that patients with hemangiomas larger than 5 cm in diameter frequently have abnormal coagulation factors with the potential to cause occurrences of blood coagulation disorder. From our investigation of coagulopathy associated with hemangiomas and the literature, we were able to conclude that hemangiomas with a diameter larger than 5 cm were defined as giant hemangiomas and that these tumors had the potential to cause blood coagulation disorder and hemangioma-related complications.

The presence of progressive abdominal pain or discomfort, spontaneous rupture, rapid growth of the tumor, and KMS are well known as hepatic hemangioma-related complications. An increasing size or intra-tumoral thrombosis or hemorrhage can cause pain or discomfort as a result of liver capsule distension [42]. Spontaneous rupture is caused by the rapid growth of the tumor leading to necrosis, increased intra-tumoral pressure with occlusion of hepatic veins, and coagulopathy [43]. The consumption of platelets and coagulation factors in addition to ongoing fibrinolysis results in intra-tumoral bleeding and the rapid enlargement of hemangiomas [44,45]. KMS is well known as a giant hepatic hemangioma characterized by thrombocytopenia and consumption coagulopathy [6,7,8]. From the above, it is suggested that hepatic hemangioma-related complications are closely related to tumor size and coagulopathy.

Although indications for surgery have traditionally included the presence of symptoms and the development of complications, which include the presence of abdominal pain or discomfort, spontaneous rupture, rapidly enlarging lesions, and KMS [41], the management of patients with asymptomatic hemangiomas remains under debate. Therefore, we attempted to elucidate the risk factors for hemangioma-related complications on the basis of echo findings, platelet counts and coagulation fibrinolytic markers (PT, fibrinogen, TAT, d-dimer, and FDP) and found that the predictive factors that require attention when evaluating patients with hemangiomas were a tumor size of >5 cm in diameter and coagulopathy, mainly the elevation of d-dimer (>5.0 µg/mL), and secondarily, the decrease in platelets and fibrinogen and the elevation of TAT and FDP. Patients who satisfy these conditions show potential for tumor enlargement and intra-tumoral thrombosis and hemorrhage, resulting in the occurrence of hemangioma-related complications. In our study, 16 patients were observed to have tumors larger than 50 mm in diameter (median size of 70.0 mm (range, 51.9–100)), although all patients, except for one patient with abdominal distension, were asymptomatic and showed almost no tendency for increased tumor size; additionally, their coagulation factors did not show a worsening trend during the observation period of up to 59 months. However, our study has some limitations regarding the length of follow up and the number of patients with giant hemangiomas, and we did not encounter any cases requiring surgery due to hemangioma-related complications; therefore, a longer follow-up period and a larger patient cohort with giant hemangiomas are needed to evaluate the exact predictive factors related to the risk accompanied by hemangiomas. However, for the management of patients with relatively large hemangiomas, we can emphasize that the follow-up tumor size measurement using US as well as the evaluation of d-dimer, platelets, and other coagulation factors (PT, fibrinogen, TAT, and FDP) during the follow-up period are indicated. In accordance with the aforementioned data, it is necessary that clinicians pay close attention during the follow-up of particular patient cohorts with the above conditions.

## 5. Conclusions

Our study was the first to demonstrate the prevalence and characteristics of hepatic hemangiomas associated with coagulopathy and elucidate the factors related to coagulopathy in patients with hemangiomas. Although further studies are required to evaluate the definite risk factors for hemangioma-related complications, we recommend that extreme care must be taken with patients that present with hemangiomas larger than 5 cm in diameter and complicated with coagulopathy, mainly the elevation of d-dimer, and secondarily, the decrease in platelets and fibrinogen and the elevation of TAT and FDP.

## Figures and Tables

**Table 1:** Laboratory findings of 281 patients with hepatic hemangiomas.

Parameters	Value
Age (years)	54 ± 15
Male/female (*n*)	98/183
Biochemistry	
Total bilirubin (mg/dL)	0.6 ± 0.2
Albumin (g/dL)	4.2 ± 0.2
ALT (U/L)	20 ± 13
GGT (U/L)	39 ± 42
ALP (U/L)	236 ± 76
BUN (mg/dL)	14.1 ± 3.7
Cr (mg/dL)	0.70 ± 0.17
LDL-chol (mg/dL)	100 ± 25
HDL-chol (mg/dL)	68 ± 18
Glucose (mg/dL)	102 ± 28
HbA1c (%)	5.5 ± 0.6
Hematology	
Hemoglobin (g/dL)	13.5 ± 1.3
WBC (/μL)	5700 ± 1600
Platelet (×10^4^/μL)	22.4 ± 5.2
Coagulation	
PT (%)	94.2 ± 12.7
Fibrinogen (mg/dL)	282 ± 74
TAT (ng/mL)	1.39 ± 0.97
D-dimer (μg/mL)	0.70 ± 0.69
FDP (μg/mL)	1.68 ± 1.04
Serology	
M2BPGi (COI)	0.55 ± 0.32
AFP (ng/mL)	3.6 ± 1.5
PIVKA-II (mAU/mL)	20.1 ± 5.8
Associated liver diseases (*n*)	
Hepatitis B	10 (3.6%)
Hepatitis C	6 (2.1%)
Autoimmune hepatitis	8 (2.8%)
Primary biliary cholangitis	2 (0.7%)
Alcoholic liver disease	9 (3.2%)
Nonalcoholic steatohepatitis	6 (2.1%)
Atherosclerotic diseases (*n*)	
Hypertension	51 (18.1%)
Dyslipidemia	27 (9.6%)
Diabetes mellitus	16 (5.7%)

**Table 2:** Ultrasonography findings of 281 patients with hepatic hemangiomas.

Characteristics	Value
Echo pattern (*n*)	
Hyperechoic	209 (74.4%)
Hypoechoic	2 (0.7%)
Isoechoic	1 (0.4%)
Mixed	69 (24.5%)
Size of hemangioma (mm)	
Small <20	190 (67.6%)
Medium 20–40	67 (23.9%)
Large >40	24 (8.5%)
Location of hemangioma (*n*)	
Right lobe	247 (87.9%)
Left lobe	32 (11.4%)
Bilateral lobes	2 (0.7%)
Number of hemangioma (*n*)	
Single	240 (85.4%)
Multiple	41 (14.6%)
Portal vein diameter (mm)	10.6 ± 2.1
Spleen index (mm^2^)	1293 ± 539

**Table 3:** (**a**) Relationship between tumor size and clinical parameters in 281 patients with hepatic hemangiomas. (**b**) Relationship between tumor size and coagulation factors in 281 patients with hepatic hemangiomas.

(**a**)				
**Parameters**	**Small (*n* = 190)**	**Medium (*n* = 67)**	**Large (*n* = 24)**	***p* Value**
Age (years)	53 ± 15	55 ± 14	60 ±15	0.0809
Male/female (*n*)	62/128	27/40	9/15	0.6721
Biochemistry				
Total bilirubin (mg/dL)	0.6 ± 0.2	0.6 ± 0.3	0.6± 0.3	0.8662
Albumin (g/dL)	4.2 ± 0.2	4.3 ± 0.2	4.1 ± 0.3	0.0013
ALT (U/L)	20 ± 14	20 ± 11	18 ± 10	0.7885
GGT (U/L)	38 ± 44	43 ± 41	32 ± 30	0.5404
ALP (U/L)	234 ± 76	237 ± 72	248 ± 88	0.6886
BUN (mg/dL)	13.8 ± 3.5	14.1 ± 3.9	16.1 ± 4.0	0.0167
Cr (mg/dL)	0.69 ± 0.16	0.71 ± 0.22	0.71 ±0.16	0.7675
LDL-chol (mg/dL)	100 ± 25	103 ±25	99 ± 19	0.6623
HDL-chol (mg/dL)	71 ± 18	65 ± 17	60 ± 17	0.0055
Glucose (mg/dL)	99 ± 25	105 ± 33	110 ± 32	0.1142
HbA1c (%)	5.4 ± 0.4	5.6 ± 1.0	5.8 ± 0.7	0.0269
Hematology				
Hemoglobin (g/dL)	13.6 ± 1.2	13.7 ±1.3	12.8 ± 1.7	0.0113
WBC (/μL)	5700 ± 1600	5600 ± 1600	5500 ± 1600	0.7754
Serology				
M2BPGi (COI)	0.47 ± 0.25	0.62 ± 0.36	0.95 ± 0.35	<0.0001
AFP (ng/mL)	3.5 ± 1.4	3.8 ± 1.6	3.3 ± 1.9	0.8175
PIVKA-Ⅱ (mAU/mL)	19.4 ± 5.4	20.8 ± 5.8	24.3 ± 6.9	0.0001
Echo pattern				<0.0001
Homogenous type (*n*)	189 (99.5%)	23 (34.3%)	0 (0%)	
Mixed type (*n*)	1 (0.5%)	44 (65.7%)	24 (100%)	
Portal vein diameter (mm)	10.1 ± 2.1	11.4 ± 1.6	12.5 ± 1.9	<0.0001
Spleen index (mm^2^)	1208 ± 505	1399 ± 499	1662 ± 696	0.0001
(**b**)				
**Parameters**	**Small (*n* = 190)**	**Medium (*n* = 67)**	**Large (*n* = 24)**	***p* Value**
Coagulation				
Platelet (×10^4^/μL)	23.1 ± 4.8	21.6 ± 5.9	18.4 ± 4.1	<0.0001
PT (%)	94.2 ± 13.0	94.2 ± 12.7	94.3 ± 10.8	0.9998
Fibrinogen (mg/dL)	290 ± 74	271 ± 71	242 ± 66	0.0019
TAT (ng/mL)	1.13 ± 0.63	1.40 ± 0.75	3.36 ± 1.36	<0.0001
D-dimer (μg/mL)	0.48 ± 0.28	0.69 ± 0.37	2.48 ± 1.01	<0.0001
FDP (μg/mL)	1.36 ± 0.32	1.57 ± 0.63	4.49 ± 1.46	<0.0001
Abnormal number of coagulation factors (*n*)	0.1 ± 0.2	0.3 ± 0.6	2.5± 1.0	<0.0001

**Table 4:** Comparison of clinical parameters among groups classified by number of abnormal coagulation factors.

Parameters	A Group (*n* = 225)	B Group (*n* = 32)	C Group (*n* = 24)	*p* Value
Age (years)	53 ± 15	55 ± 14	59 ±14	0.1546
Male/female (*n*)	74/151	13/19	11/13	0.5881
Biochemistry				
Total bilirubin (mg/dL)	0.6 ± 0.3	0.6 ± 0.2	0.6 ± 0.3	0.9753
Albumin (g/dL)	4.3 ± 0.2	4.2 ± 0.3	4.1 ± 0.3	0.0050
ALT (U/L)	19 ± 12	23 ± 16	20 ± 10	0.2543
GGT (U/L)	40 ± 45	28 ± 26	36 ± 31	0.2918
ALP (U/L)	237 ± 74	222 ± 83	247 ± 86	0.4305
BUN (mg/dL)	13.8 ± 3.6	15.1 ± 4.8	15.1 ± 2.8	0.0555
Cr (mg/dL)	0.69 ± 0.16	0.74 ± 0.25	0.72 ±0.18	0.2185
LDL-chol (mg/dL)	101 ± 25	102 ±20	97 ± 25	0.7537
HDL-chol (mg/dL)	70 ± 18	64 ± 18	58 ± 16	0.0020
Glucose (mg/dL)	101 ± 27	100 ± 25	113 ± 35	0.0949
HbA1c (%)	5.5 ± 0.7	5.6 ± 0.4	5.8 ± 0.7	0.0519
Hematology				
Hemoglobin (g/dL)	13.6 ± 1.3	13.4 ±1.4	13.1 ± 1.8	0.2551
WBC (/μL)	5700 ± 1600	5300 ± 1500	5700 ± 1700	0.4135
Serology				
M2BPGi (COI)	0.48 ± 0.24	0.74 ± 0.42	0.96 ± 0.40	<0.0001
AFP (ng/mL)	3.6 ± 1.5	3.5 ± 1.1	3.4 ± 1.9	0.8175
PIVKA-Ⅱ (mAU/mL)	19.7 ± 5.6	19.5 ± 4.6	25.0 ± 6.7	0.0001
Echo findings				
Tumor size (mm)	14.0 ± 6.7	24.2 ± 11.8	59.5 ± 21.7	<0.0001
Echo pattern				<0.0001
Homogeneous type (*n*)	198 (88.0%)	14 (43.8%)	0 (0%)	
Mixed type (*n*)	27 (12.0%)	18 (56.2%)	24 (100%)	
Portal vein diameter (mm)	10.3 ± 2.0	10.5 ± 1.8	13.1 ± 1.7	<0.0001
Spleen index (mm^2^)	1236 ± 501	1394 ± 589	1703 ± 599	0.0002
Associated liver diseases (*n*)	27 (12.0%)	8 (24.2%)	6 (26.1%)	0.0487

A group: normal values of all of PLT, fibrinogen, TAT, D-dimer, and FDP. B group: abnormal values of one of PLT, fibrinogen, TAT, and D-dimer or FDP. C group: abnormal values of 2 or more of PLT, fibrinogen, TAT, D-dimer, or FDP.

**Table 5:** Occurrence rate of abnormal coagulation factors in 281 patients with hepatic hemangiomas.

Abnormal Factor	Number of Patients	Abnormal Number	Number of Patients
Platelet	5 (1.8%)	1 factor	32 (11.4%)
PT	2 (0.7%)	2 factors	13 (4.6%)
Fibrinogen	7 (2.5%).	3 factors	8 (2.8%)
TAT	23 (8.2%)	4 factors	2 (0.7%)
D-dimer	45 (16.0%)	5 factors	1 (0.4%)
FDP	13 (4.6%)	6 factors	0 (0%)

**Table 6:** Multiple regression analysis of variables associated with numbers of abnormal coagulation factors in 281 patients with hepatic hemangiomas.

Variables	Β	SE (β)	*t* Value	*p* Value
Echo pattern (*n*)	0.04045	0.10224	0.39560	0.6927
Tumor size (mm)	0.03637	0.00294	12.3662	0.0000
Portal vein diameter (mm)	−0.0077	0.01657	0.46583	0.6417
Spleen index (mm^2^)	0.00009	0.00006	1.55899	0.1202
PIVKA-Ⅱ (mAU/mL)	0.00326	0.00550	0.59143	0.5547
M2BPGi (COI)	0.29675	0.12761	2.32544	0.0208
Albumin (g/dL)	−0.1489	0.13229	1.12550	0.2614
AST (U/L)	0.00437	0.00354	1.23349	0.2185
BUN (mg/dL)	0.00146	0.00842	0.17359	0.8623
HDL-chol (mg/dL)	−0.0042	0.00181	2.31312	0.0215
Glucose (mg/dL)	−0.0012	0.00116	1.03497	0.3016

**Table 7:** Comparison of clinical parameters in patients with and without chronic liver disease as an underlying disease.

Parameters	Liver Disease (+) *n* = 41	Liver Disease (−) *n* = 240	*p* Value
Age (years)	60 ± 11	53 ± 15	0.0009
Male/female (*n*)	17/24	81/159	0.6221
Biochemistry			
Total bilirubin (mg/dL)	0.6 ± 0.3	0.6 ± 0.2	0.3781
Albumin (g/dL)	4.2 ± 0.3	4.2 ± 0.2	0.0892
ALT (U/L)	30 ± 24	18 ± 7	0.0049
GGT (U/L)	65 ± 72	34 ± 33	0.0109
ALP (U/L)	252 ± 96	233 ± 72	0.2251
BUN (mg/dL)	14.0 ± 3.9	14.1 ± 3.7	0.9705
Cr (mg/dL)	0.72 ± 0.17	0.69 ± 0.17	0.2753
LDL-chol (mg/dL)	94 ± 27	101 ± 24	0.1206
HDL-chol (mg/dL)	68 ± 24	69 ± 17	0.8066
TG (mg/dL)	109 ± 67	96 ± 56	0.2317
Glucose (mg/dL)	107 ± 24	101 ± 28	0.1661
HbA1c (%)	5.5 ± 0.4	5.5 ± 0.7	0.8694
Hematology			
Hemoglobin (g/dL)	13.7 ± 1.4	13.5 ± 1.3	0.4266
WBC (/μL)	5300 ± 1600	5700 ± 1600	0.1061
Platelet (×10^4^/μL)	19.9 ± 5.4	22.8 ± 5.1	0.0023
Cagulation			
PT (%)	93.8 ± 12.4	94.3 ± 12.4	0.8359
Fibrinogen (mg/dL)	261 ± 67	285 ± 75	0.0289
TAT (ng/mL)	1.78 ± 1.43	1.32 ± 0.85	0.0668
D-dimer (μg/mL)	0.88 ± 0.72	0.67 ± 0.68	0.0813
FDP (μg/mL)	1.91 ± 1.22	1.64 ± 1.01	0.1865
Serology			
M2BPGi (COI)	0.78 ± 0.39	0.51 ± 0.29	0.0001
AFP (ng/mL)	3.4 ± 1.9	3.6 ± 1.4	0.4874
PIVKA-II (mAU/mL)	21.4 ± 5.8	19.9 ± 5.8	0.1489
Tumor size (mm)	23.0 ± 16.8	18.2 ± 15.4	0.0956
Portal vein diameter (mm)	11.4 ± 2.3	10.4 ±2.1	0.0110
Spleen index (mm^2^)	1392 ± 520	1276 ±541	0.1973
Abnormal number of coagulation factors (*n*)	0.7 ± 1.3	0.3 ± 0.7	0.0607

**Table 8:** Comparison of clinical parameters in patients with and without atherosclerotic disease *.

Parameters	Atherosclerotic Diseases (+) *n* = 69	Atherosclerotic Diseases (−) *n* = 212	*p* Value
Age (years)	64 ± 12	50 ± 14	0.0000
Male/female (*n*)	28/41	69/143	0.6464
Biochemistry			
Total bilirubin (mg/dL)	0.6 ± 0.3	0.6 ± 0.2	0.2626
Albumin (g/dL)	4.2 ± 0.3	4.2 ± 0.2	0.4936
ALT (U/L)	21 ± 19	20 ± 10	0.4779
GGT (U/L)	43 ± 56	37 ± 36	0.4384
ALP (U/L)	244 ± 93	233 ± 69	0.3656
BUN (mg/dL)	15.9 ± 4.2	13.5 ± 3.3	0.0000
Cr (mg/dL)	0.77 ± 0.23	0.67 ± 0.14	0.0015
LDL-chol (mg/dL)	97 ± 25	101 ± 24	0.2419
HDL-chol (mg/dL)	61 ± 16	71 ± 18	0.0001
TG (mg/dL)	118 ± 65	91 ± 53	0.0030
Glucose (mg/dL)	117 ± 45	97 ± 16	0.0006
HbA1c (%)	5.9 ± 1.1	5.4 ± 0.3	0.0001
Hematology			
Hemoglobin (g/dL)	13.6 ± 1.3	13.5 ± 1.3	0.4661
WBC (/μL)	5800 ± 1500	5600 ± 1600	0.3363
Platelet (x10^4^/μL)	22.4 ± 6.4	22.3 ± 4.8	0.8882
Coagulation			
PT (%)	94.0 ± 12.3	94.3 ± 13.0	0.8667
Fibrinogen (mg/dL)	278 ± 67	283 ± 76	0.6725
TAT (ng/mL)	1.45 ± 1.08	1.37 ± 0.93	0.5593
D-dimer (μg/mL)	0.89± 0.86	0.63 ± 0.61	0.0426
FDP (μg/mL)	1.82 ± 1.28	1.63 ± 0.95	0.2770
Serology			
M2BPGi (COI)	0.62 ± 0.37	0.53 ± 0.30	0.0249
AFP (ng/mL)	3.8 ± 1.6	3.5 ± 1.4	0.1115
PIVKA-II (mAU/mL)	21.8 ± 6.4	19.6 ± 5.5	0.0086
Tumor size (mm)	20.9 ± 17.5	18.3 ± 15.1	0.2547
Portal vein diameter (mm)	10.8 ± 2.0	10.5 ±2.2	0.2269
Spleen index (mm^2^)	1178 ± 430	1330 ±566	0.0210
Abnormal number of coagulation factors (*n*)	0.5 ± 1.0	0.3 ± 0.8	0.2254

* Atherosclerotic diseases are hypertension, dyslipidemia and diabetes mellitus.

## Data Availability

The data presented in this study are available on request from the corresponding author.

## Abbreviations

PT	Prothrombin time
TAT	Thrombin–antithrombin III complex
FDP	Fibrin and fibrinogen degradation products
US	Ultrasonography
CT	Computed tomography
PIVKA-II	Protein induced by vitamin K absence-II
M2BPGi	Mac-2 binding protein glycosylation isomer
KMS	Kasabach–Merritt syndrome
